# Association between CALLY index and all-cause mortality in patients with rheumatoid arthritis

**DOI:** 10.3389/fnut.2026.1829076

**Published:** 2026-06-16

**Authors:** Tong Zhang, Liang V. Tang

**Affiliations:** 1 Institute of Hematology, Union Hospital, Tongji Medical College, Huazhong University of Science and Technology, Wuhan, China; 2 Key Lab of Molecular Biological Targeted Therapies of the Ministry of Education, Union Hospital, Tongji Medical College, Huazhong University of Science and Technology, Wuhan, China

**Keywords:** all-cause mortality, CALLY index, prognosis, rheumatoid arthritis, UK biobank

## Abstract

**Purpose:**

The CALLY index reflects immune, inflammatory, and nutritional status, but its prognostic role in rheumatoid arthritis (RA) remains uncertain. We investigated whether the CALLY index was independently associated with all-cause mortality in patients with RA, and further examined its nonlinear pattern, and consistency across clinical subgroups.

**Methods:**

We included 5,626 participants with RA from the UK Biobank, with a median follow-up of 15.6 years. Multivariable Cox regression models were used to assess the association between the CALLY index and all-cause mortality. Restricted cubic splines were used to explore potential nonlinearity. Predictive performance was evaluated using time-dependent ROC analysis, IDI, and NRI. Subgroup and sensitivity analyses were performed to test the robustness of the findings.

**Results:**

A higher CALLY index was independently associated with a lower risk of all-cause mortality in RA patients. This association showed a nonlinear inverse dose–response pattern, with an estimated inflection point of 1.054. The CALLY index showed better reclassification than its individual components and achieved non-inferior predictive performance compared with the combined three-biomarker model. The association was broadly consistent in clinical subgroups and stable in sensitivity analyses.

**Conclusion:**

The CALLY index may serve as a simple, low-cost biomarker for long-term mortality risk stratification in patients with RA.

## Introduction

Rheumatoid arthritis (RA) is a chronic autoimmune disease characterized by persistent systemic inflammation (1, 2). Compared with the general population, patients with RA have a higher long-term risk of death, mainly due to cardiovascular disease, malignancy, and inflammation-related organ damage (3–7). Better prognostic assessment is important for clinical follow-up and management of these patients. Clinically, C-reactive protein (CRP), albumin, and lymphocyte levels are commonly used to reflect inflammatory burden, nutritional and immune status. However, each marker only reflects limited biological profile. They may not adequately represent the complicate interaction between inflammation, nutrition, and immunity in RA when used alone.

The CALLY index combines albumin, lymphocyte level, and CRP together. It has been reported as a prognostic marker in several studies (8–10). Nevertheless, several problems remain unresolved. First, it is unclear whether the prognostic association is also present in European RA populations, who may possess different genetic background, lifestyle, comorbidity patterns, and medication using. Second, most available studies have focused on association rather than on whether CALLY adds information than its components. Third, evidence is still limited on the nonlinear pattern, the appropriate cut-off point, and the consistency of the association across RA subgroups.

Based on a large RA cohort from the UK Biobank (UKB), we proposed three hypotheses. First, higher CALLY levels would be associated with lower all-cause mortality, with a potentially nonlinear dose–response pattern. Second, as a composite indicator, the CALLY index would offer better predictive and reclassification performance than its individual components. Finally, the association would remain stable across clinically subgroups and in sensitivity analyses. Then, we evaluated baseline characteristics, the association between CALLY and mortality, predictive performance, nonlinear patterns, subgroup heterogeneity, and added clinical utility in the UKB RA cohort. The aim was to clarify whether the CALLY index can serve as a practical and low-cost tool for mortality risk assessment in patients with RA.

## Methods

### Study design and data source

Raw data were extracted from UKB (11). Ethical approval was granted by the Northwest Multi-Center Research Ethics Committee, and all enrolled participants gave written informed consent. The approved application number from the UKB is 982,980.

### Study population

Firstly, based on International Classification of Diseases (ICD)-10 codes and algorithm-defined diagnosis, participants in the UKB with a baseline diagnosis of RA were enrolled. Then, participants who had incomplete data in the CALLY index components or follow-up outcomes were excluded. Finally, those who were pregnant at baseline were excluded.

### Definition and measurement of variables

We calculated the CALLY index by integrating albumin (g/dL), lymphocyte level (10^9/L), and CRP (mg/L) levels using the following formula: (albumin × lymphocyte level)/CRP × 10^4. The original CALLY index was processed via natural logarithmic transformation (lnCALLY). This transformation was performed to correct skewed data distribution, and reduce the influence of outliers. Quartile stratification was performed according to the lnCALLY index, and Q1 was the reference group in Cox regression analysis. The primary study outcome was all-cause mortality. Deaths were defined using UKB registry data. Follow-up time was calculated from baseline enrolment to death or the final observational time point.

### Missing data handling and cox regression modeling

Multiple Imputation by Chained Equations (MICE) was employed to fill missing baseline covariates. Results from imputed datasets were used in Cox regression models to explore the prognostic association. Model 0 was a univariate regression analysis of lnCALLY, yielding crude hazard ratios (HRs) and 95% confidence intervals (CIs).

To control for potential confounding while minimizing arbitrary covariate selection, we adopted a sequential adjustment strategy guided by an established epidemiological hierarchy. Firstly, age and gender were accounted for in Model 1. Model 2 further adjusted for socioeconomic and lifestyle factors. Model 3 additionally included major clinical comorbidities to reduce potential bias from competing mortality risks. Model 4 was the fully adjusted model, with adjustment for biochemical markers.

To further support the covariate structure and reduce the risk of overfitting, we performed backward stepwise selection based on the Akaike Information Criterion (AIC). Moreover, adjusted Variance inflation factors (VIFs) were assessed to confirm the absence of substantial multicollinearity among the predictive variables.

### Time-dependent receiver operating characteristic analysis

Time-dependent ROC analysis with AUC metrics (timeROC package) was performed to compare model predictive efficacy. Additionally, the survIDINRI R package was utilized to compute continuous NRI and IDI for the evaluation of reclassification performance. A perturbation resampling method (300 iterations) was employed to estimate the 95% CIs and *p* values for NRI and IDI.

### Non-linear relationship and survival analysis

The continuous relationship between CALLY and all-cause mortality was evaluated over the full follow-up duration. A four-knot restricted cubic spline (RCS) was fitted to the fully adjusted Cox model to detect nonlinear trends. Sensitivity analyses using three and five knots were also conducted. Then, a maximum likelihood-based recursive grid-search algorithm was employed on the entire dataset to determine the optimal exploratory cut-off. A two-piecewise Cox proportional hazards model was then constructed to quantify the threshold effect. Likelihood ratio test (LRT) was used to compare the fit of the piecewise model with the linear model. Finally, survival curves were drawn using the Kaplan–Meier method.

### Subgroup analysis

Within different sociodemographic, lifestyle, and clinical subgroups, HRs and 95% CIs were calculated using the same adjusted Cox regression models as the primary analysis.

### Sensitivity analysis

Sensitivity analyses were carried out to verify the reliability of the main results. First, participants without imputed data were used to validate the primary findings. Second, to assess the potential influence of extreme laboratory values, Cox regression models were repeated after excluding participants with lymphocyte count > 5 × 10^9/L and after excluding values above Q3 + 3IQR. Third, to reduce the risk of reverse causality, participants who died within the first year of follow-up were excluded. Lastly, Cox regression models were reanalyzed after the CALLY index was stratified into tertiles.

### Statistical analysis

To evaluate potential selection bias, baseline characteristics were compared between participants included in the analysis and those excluded because of missing relevant data. Baseline balance was assessed using standardized mean differences (SMDs). An SMD < 0.1 was considered as acceptable to neglect between-group difference.

Continuous variables were summarized as medians with interquartile ranges (IQRs) and compared using the Mann–Whitney U test. Categorical variables were reported as numbers and proportions, and differences between groups were evaluated using the Pearson chi-square test.

Schoenfeld residuals were applied to test the proportional hazards assumption for Cox regression models. The proportional hazards assumption was assessed in the fully adjusted model at both the global and covariate-specific levels. Results were shown in scaled residual plots.

Statistical analyses were performed in R (version 2025.09.2 + 418). The significance level was set at *p* < 0.05 unless otherwise indicated.

## Results

### Baseline profile analysis of the study participants

**Figure 1:**
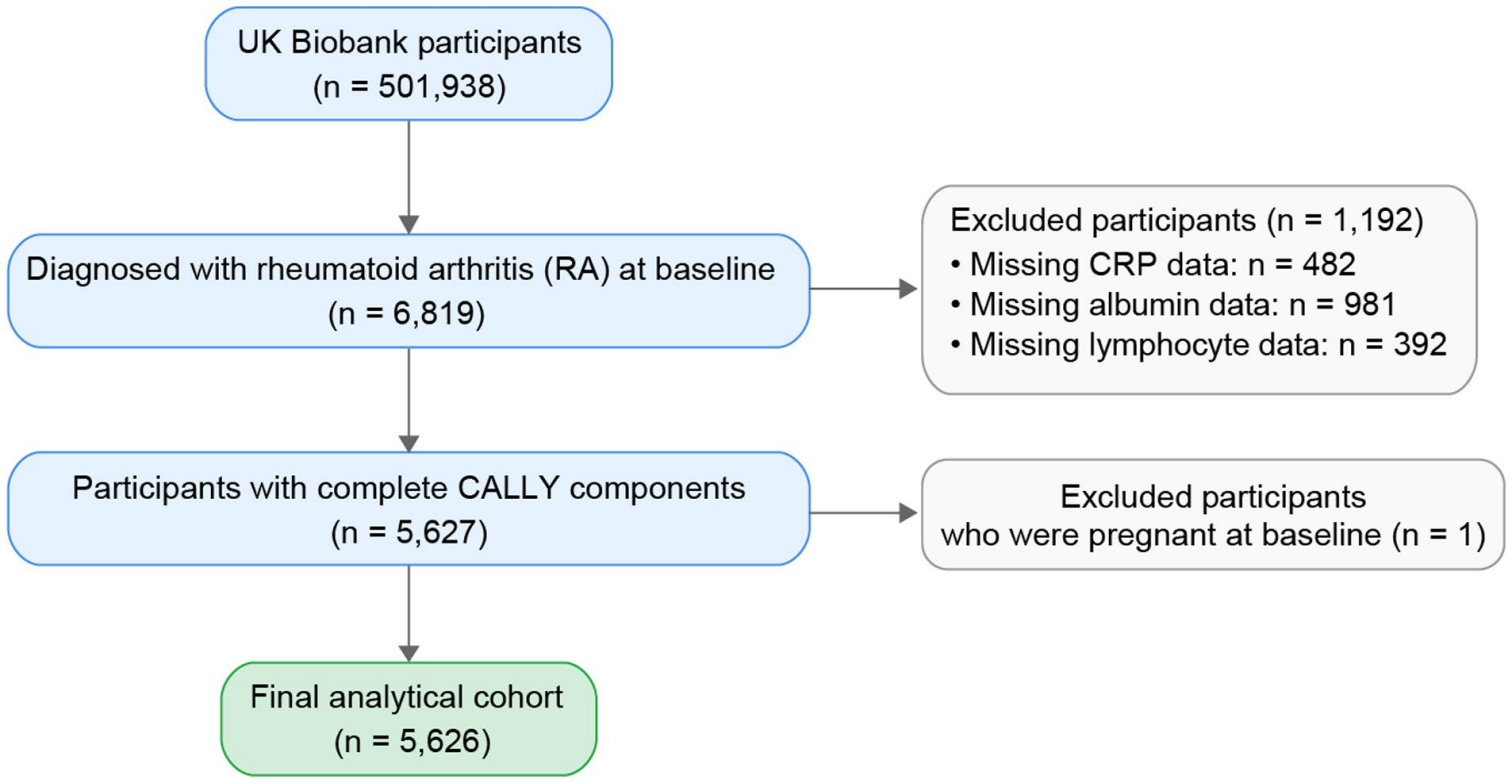
Participant screening and inclusion flowchart. The flowchart outlining the enrollment process for UKB participants. The initial cohort consisted of 501,938 participants, which was then restricted to those with a baseline rheumatoid arthritis (RA) diagnosis (6,819 individuals). A total of 1,192 participants were subsequently excluded due to missing baseline data required for the CALLY index calculation. Notably, the missing data counts for C-reactive protein (CRP), albumin, and lymphocytes are non-mutually exclusive, as some participants lacked data for multiple biomarkers simultaneously. Finally, after excluding one participant due to pregnancy status, the final analytical cohort yielded 5,626 participants.

After exclusions, 5,626 individuals with RA were enrolled for the final analysis (Figure 1). The cohort was followed for a median of 15.6 years, during which 1,179 all-cause death events were documented. In the comparison of the included cohort (*n* = 5,626) and excluded cohort (*n* = 1,193), all baseline variables yielded SMD below 0.1, suggesting well-balanced baseline characteristics across groups and negligible selection bias (Supplementary Table S2).

Decedents were older and more likely to be male than survivors. They also had higher smoking rates and a greater prevalence of cancer and cardiovascular comorbidities (Supplementary Table S3). Additionally, they showed a less favorable metabolic, inflammatory, and organ function profile, together with significantly lower median lnCALLY scores (*p* < 0.001). When participants were stratified by lnCALLY quartiles, all-cause mortality showed a clear inverse gradient, falling from 30.49% in the lowest quartile to 15.21% in the highest quartile (*p* < 0.001; Table 1). Higher lnCALLY quartiles were characterized by a lower prevalence of obesity, better educational attainment, and a reduced cancer burden. Similar gradients were observed for the biomarkers that constitute the CALLY index. As lnCALLY quartiles increased, CRP levels declined markedly, whereas serum albumin levels and lymphocyte counts rose steadily (all *p* < 0.001).

**Table 1:** Characteristics of study participants according to lnCALLY quartiles.

Characteristic	lnCALLY Q1 [−3.87,0.187] *n* = 1,407	lnCALLY Q2 (0.187,1.05) *n* = 1,406	lnCALLY Q3 (1.05,1.92) *n* = 1,406	lnCALLY Q4 (1.92,4.79) *n* = 1,407	*p*
Status = Dead (%)	429.00 (30.49%)	294.00 (20.91%)	242.00 (17.21%)	214.00 (15.21%)	<0.001
Follow-up time [median (IQR)]	15.41 [14.00, 16.34]	15.69 [14.64, 16.54]	15.74 [14.71, 16.50]	15.72 [14.77, 16.49]	<0.001
Age [median (IQR)]	61.00 [55.00, 65.00]	61.00 [55.00, 65.00]	60.00 [54.00, 65.00]	60.00 [54.00, 64.00]	<0.001
Gender = Male (%)	465.00 (33.05%)	426.00 (30.30%)	453.00 (32.22%)	437.00 (31.06%)	0.408
Ethnicity = White (%)	1,335.00 (94.88%)	1,329.00 (94.52%)	1,328.00 (94.45%)	1,319.00 (93.75%)	0.613
BMI					<0.001
Normal (≥18.5 < 25)	341.00 (24.24%)	293.00 (20.84%)	347.00 (24.68%)	569.00 (40.44%)	
Obese (≥30)	566.00 (40.23%)	575.00 (40.90%)	443.00 (31.51%)	260.00 (18.48%)	
Overweight (≥25 < 30)	488.00 (34.68%)	531.00 (37.77%)	608.00 (43.24%)	563.00 (40.01%)	
Underweight (<18.5 ~ 1)	12.00 (0.85%)	7.00 (0.50%)	8.00 (0.57%)	15.00 (1.07%)	
Education					<0.001
College	260.00 (18.48%)	281.00 (19.99%)	287.00 (20.41%)	405.00 (28.78%)	
Other	651.00 (46.27%)	698.00 (49.64%)	709.00 (50.43%)	676.00 (48.05%)	
Unknown	496.00 (35.25%)	427.00 (30.37%)	410.00 (29.16%)	326.00 (23.17%)	
Smoking status					0.008
Current	163.00 (11.58%)	173.00 (12.30%)	178.00 (12.66%)	172.00 (12.22%)	
Never	627.00 (44.56%)	634.00 (45.09%)	705.00 (50.14%)	687.00 (48.83%)	
Previous	617.00 (43.85%)	599.00 (42.60%)	523.00 (37.20%)	548.00 (38.95%)	
Cancer = Yes (%)	329.00 (23.38%)	294.00 (20.91%)	280.00 (19.91%)	252.00 (17.91%)	0.004
CHD^1^ = Yes (%)	118.00 (8.39%)	119.00 (8.46%)	118.00 (8.39%)	101.00 (7.18%)	0.538
CRP (median [IQR])	11.63 [7.57, 19.41]	4.01 [2.92, 5.46]	1.90 [1.47, 2.45]	0.73 [0.49, 1.07]	<0.001
Lymphocyte [median (IQR)]	1.50 [1.14, 1.90]	1.72 [1.39, 2.20]	1.86 [1.48, 2.30]	1.98 [1.60, 2.47]	<0.001
Albumin [median (IQR)]	42.96 [40.95, 44.91]	44.29 [42.60, 45.95]	44.75 [43.13, 46.59]	45.37 [43.81, 47.17]	<0.001
ALT [median (IQR)]	18.92 [14.33, 26.30]	20.76 [15.93, 27.81]	20.88 [15.99, 28.68]	19.55 [14.99, 26.20]	<0.001
AST [median (IQR)]	24.00 [20.30, 29.10]	25.00 [21.30, 30.10]	25.20 [21.60, 30.00]	24.50 [20.90, 28.90]	<0.001
LDL [median (IQR)]	3.35 [2.79, 3.95]	3.55 [2.92, 4.10]	3.47 [2.89, 4.10]	3.43 [2.83, 4.07]	<0.001
HbA1c [median (IQR)]	35.50 [32.30, 39.00]	35.60 [32.40, 38.80]	35.50 [32.70, 38.40]	34.90 [32.30, 37.60]	<0.001
Creatinine [median (IQR)]	67.10 [57.70, 79.50]	67.40 [59.00, 78.00]	66.95 [58.30, 77.80]	66.70 [58.80, 76.30]	<0.001

^1^CHD, coronary heart disease.

### Mortality predictive performance of the CALLY index

Collinearity diagnostics showed no substantial multicollinearity among the covariates included in the multivariable Cox regression models (Supplementary Table S4). Regarding the proportional hazards assumption, no evident violation was observed for either the overall model or the CALLY index within the primary 5-year clinical prognostic window (*p* = 0.757 and *p* = 0.118, respectively). However, when extended to the full follow-up term, the assumption was not met (Global *p* = 0.012, CALLY *p* = 0.017), reflecting an expected time-dependent attenuation of baseline biomarker predictive capacity. These findings were further confirmed by graphical assessments (Supplementary Table S5; Supplementary Figure S1).

In the Cox regression analyses, higher CRP levels were consistently associated with an increased risk of all-cause mortality, whereas higher albumin concentrations and lnCALLY index values were associated with lower mortality risk (Table 2). Similar patterns were observed when these biomarkers were analyzed by quartiles. Mortality risk decreased progressively across increasing quartiles of lymphocyte count, albumin concentration, and the lnCALLY index, but increased across higher CRP quartiles (all *p* for trend < 0.001). Notably, the composite lnCALLY index showed a stable graded inverse association with mortality across all adjusted models.

**Table 2:** Relationships of lymphocyte level, CRP, and albumin with all-cause mortality in RA patients.

Variable	Model 0 HR (95% CI)	Model 1 HR (95% CI)	Model 2 HR (95% CI)	Model 3 HR (95% CI)	Model 4 HR (95% CI)
Lymphocyte(continuous)	0.995 (0.918–1.078)	1.036 (0.966–1.111)	0.963 (0.888–1.044)	0.956 (0.881–1.038)	0.963 (0.886–1.045)
Lymphocyte(4-class)					
[0.22,1.4]	Ref	Ref	Ref	Ref	Ref
(1.4,1.78)	0.679 (0.577–0.799)	0.719 (0.611–0.846)	0.700 (0.595–0.824)	0.698 (0.593–0.821)	0.697 (0.591–0.821)
(1.78,2.22)	0.700 (0.599–0.818)	0.766 (0.656–0.896)	0.709 (0.606–0.830)	0.702 (0.600–0.822)	0.703 (0.600–0.823)
(2.22,30.2)	0.786 (0.675–0.915)	0.883 (0.758–1.029)	0.742 (0.634–0.869)	0.727 (0.620–0.851)	0.725 (0.618–0.851)
*p* for trend	0.001	0.089	<0.001	<0.001	<0.001
CRP (continuous)	1.023 (1.018–1.028)	1.021 (1.016–1.026)	1.019 (1.014–1.024)	1.019 (1.014–1.024)	1.018 (1.013–1.023)
CRP (4-class)					
[0.09,1.25]	Ref	Ref	Ref	Ref	Ref
(1.25,2.7)	1.149 (0.957–1.380)	1.038 (0.864–1.246)	1.016 (0.844–1.223)	1.032 (0.857–1.241)	1.051 (0.874–1.265)
(2.7,6.22)	1.495 (1.256–1.778)	1.409 (1.184–1.676)	1.361 (1.139–1.626)	1.377 (1.153–1.645)	1.403 (1.174–1.675)
(6.22,77.7)	2.090 (1.773–2.464)	1.959 (1.661–2.310)	1.820 (1.538–2.155)	1.850 (1.562–2.190)	1.833 (1.548–2.171)
*P* for trend	<0.001	<0.001	<0.001	<0.001	<0.001
Albumin (continuous)	0.890 (0.873–0.908)	0.895 (0.878–0.913)	0.901 (0.884–0.919)	0.902 (0.884–0.920)	0.909 (0.891–0.928)
Albumin (4-class)					
[27.1,42.6]	Ref	Ref	Ref	Ref	Ref
(42.6,44.4)	0.633 (0.545–0.736)	0.633 (0.545–0.735)	0.631 (0.543–0.733)	0.639 (0.550–0.743)	0.682 (0.586–0.793)
(44.4,46.3)	0.562 (0.482–0.656)	0.574 (0.492–0.671)	0.590 (0.505–0.689)	0.590 (0.505–0.689)	0.624 (0.534–0.731)
(46.3,55.4)	0.447 (0.379–0.528)	0.476 (0.402–0.562)	0.493 (0.417–0.583)	0.491 (0.415–0.581)	0.518 (0.437–0.614)
*P* for trend	<0.001	<0.001	<0.001	<0.001	<0.001
lnCALLY (continuous)	0.775 (0.742–0.809)	0.795 (0.761–0.830)	0.800 (0.765–0.836)	0.799 (0.765–0.835)	0.806 (0.771–0.842)
lnCALLY (4-class)					
[−3.87,0.187]	Ref	Ref	Ref	Ref	Ref
(0.187,1.05)	0.640 (0.552–0.743)	0.651 (0.561–0.755)	0.657 (0.566–0.763)	0.647 (0.557–0.750)	0.672 (0.578–0.780)
(1.05,1.92)	0.512 (0.437–0.599)	0.526 (0.450–0.616)	0.537 (0.458–0.629)	0.526 (0.449–0.617)	0.536 (0.457–0.629)
(1.92,4.79)	0.449 (0.381–0.529)	0.488 (0.414–0.575)	0.502 (0.425–0.593)	0.494 (0.418–0.584)	0.508 (0.430–0.600)
*p* for trend	<0.001	<0.001	<0.001	<0.001	<0.001

To assess whether lnCALLY added predictive information beyond its individual components, we compared its discrimination and reclassification performance using time-dependent ROC analysis, IDI, and continuous NRI (Supplementary Table S6). The composite index outperformed lymphocyte count alone, showing a higher 5-year AUC (ΔAUC = 0.100; *p* < 0.001) and better risk reclassification (NRI = 0.267; 95% CI, 0.177 to 0.330; *p* < 0.001). When comparing to CRP, lnCALLY showed only a modest difference in AUC, but still improved mortality risk reclassification at 3 years (NRI = 0.233; *p* < 0.001) and 5 years (NRI = 0.173; *p* < 0.001). Its performance was also non-inferior to albumin, with an improvement in 5-year discrimination that did not reach statistical significance (ΔAUC = 0.044; *p* = 0.075).

The CALLY-based model was further compared with a multi-component model that included CRP, albumin, and lymphocyte count as separate predictors. At 5 years, the time-dependent AUC was 0.746 for the CALLY model and 0.742 for the multi-component model, indicating nearly identical discrimination. Consistent with this finding, reclassification analyses showed no significant difference between the two models in IDI (0.001; 95% CI, −0.019 to 0.004; *p* = 0.930) or continuous NRI (0.048; 95% CI, −0.044 to 0.141; *p* = 0.352). Thus, the CALLY index appeared to achieve predictive performance comparable to that of the three separate biomarkers.

### RCS analysis and survival analysis

**Figure 2:**
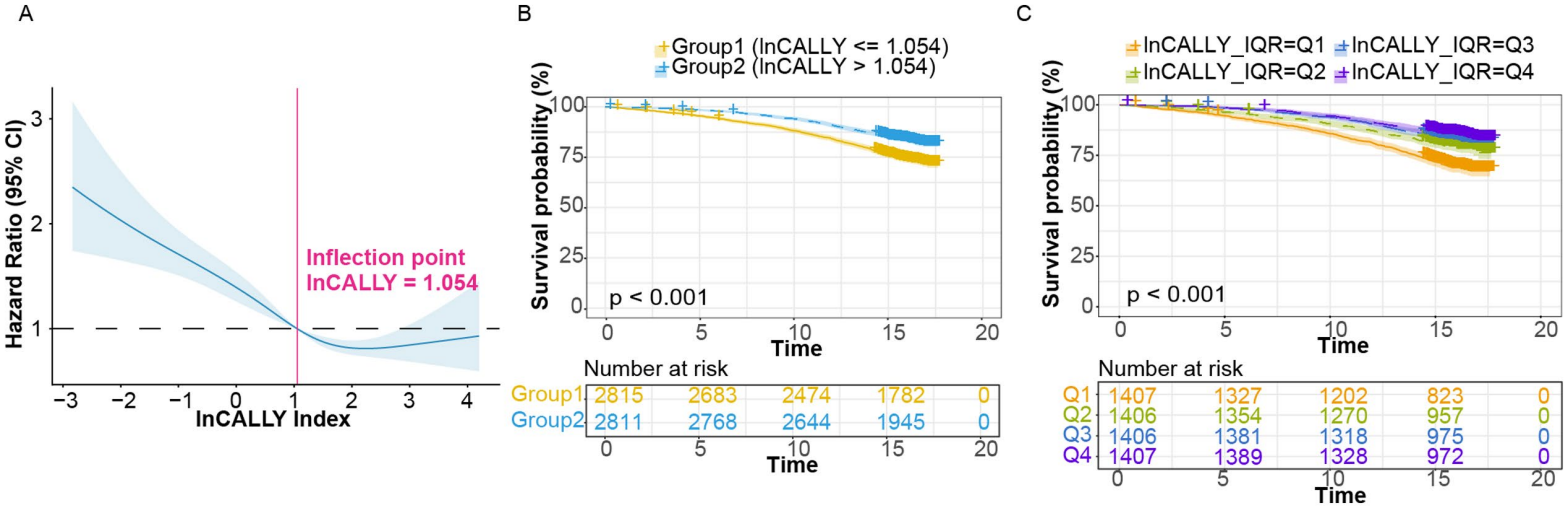
Dose–response relationship and Kaplan–Meier survival analyses of the lnCALLY index for all-cause mortality. **(A)** Restricted cubic spline (RCS) curve demonstrating the non-linear association between the continuous lnCALLY index and the hazard ratio of all-cause mortality. The estimated hazard ratio is shown by the solid blue line, with the shaded area referring to the 95% CI. The horizontal dashed line represents the reference hazard ratio of 1.0. A significant inflection point was identified at lnCALLY = 1.054 (indicated by the vertical pink line). **(B)** Kaplan–Meier survival curves stratifying patients into two groups based on the RCS-derived inflection point: Group 1 (<=1.054) and Group 2 (>1.054). **(C)** Kaplan–Meier survival curves stratifying patients into four risk categories based on the quartiles (Q1-Q4) of the lnCALLY index. In panels B and C, the tables below the curves detail the number of patients at risk at specified time intervals.

An exploratory lnCALLY cut-off of 1.054 was identified through grid-search analysis and RCS modeling (Figure 2A). This data-derived inflection point remained stable in sensitivity analyses using different knot placements (Supplementary Figure S2). In the lower range of lnCALLY values (<= 1.054), each 1-unit increase was associated with a 22.8% reduction in all-cause mortality risk (HR, 0.772; 95% CI, 0.721 to 0.826; *p* < 0.001). Above this threshold, the inverse association persisted but was weaker (HR, 0.877; 95% CI, 0.784 to 0.981; *p* = 0.021). However, the likelihood ratio test did not show a statistically significant improvement in model fit for the piecewise model over the linear model (*p* = 0.110), suggesting that the overall association was largely continuous. Even so, the cut-off of 1.054 may still provide a useful exploratory reference for stratifying patients into different risk groups.

To visualize the survival differences, Kaplan–Meier analyses were performed after stratifying the RA cohort by clinically relevant lnCALLY thresholds. Using the RCS-derived inflection point of 1.054 as the first cut-off, participants were divided into lower and higher lnCALLY groups (Figure 2B). The two survival curves separated clearly over follow-up, with a significant log-rank result (*p* < 0.001). Patients with lnCALLY values above 1.054 showed consistently better survival probability than those at or below this threshold.

To present the prognostic gradient in a more clinically familiar way, participants were also grouped into quartiles according to baseline lnCALLY levels. The Kaplan–Meier curves showed a stepwise improvement in survival from Q1 to Q4, with a significant overall difference across groups (log-rank *p* < 0.0001; Figure 2C). Pairwise comparisons supported this pattern (Supplementary Table S7). Participants in the lowest quartile had significantly poorer survival than those in each of the other quartiles (all *p* < 0.001). However, the survival advantage did not continue to increase indefinitely. No significant difference was observed between Q3 and Q4 (*p* = 0.952), suggesting a plateau at higher lnCALLY levels. This pattern was consistent with the RCS analysis and supports the idea that the major risk separation occurs around the Q2 to Q3 boundary, close to the identified inflection point of 1.054.

### Subgroup stratification of the prognostic association of lnCALLY

**Figure 3:**
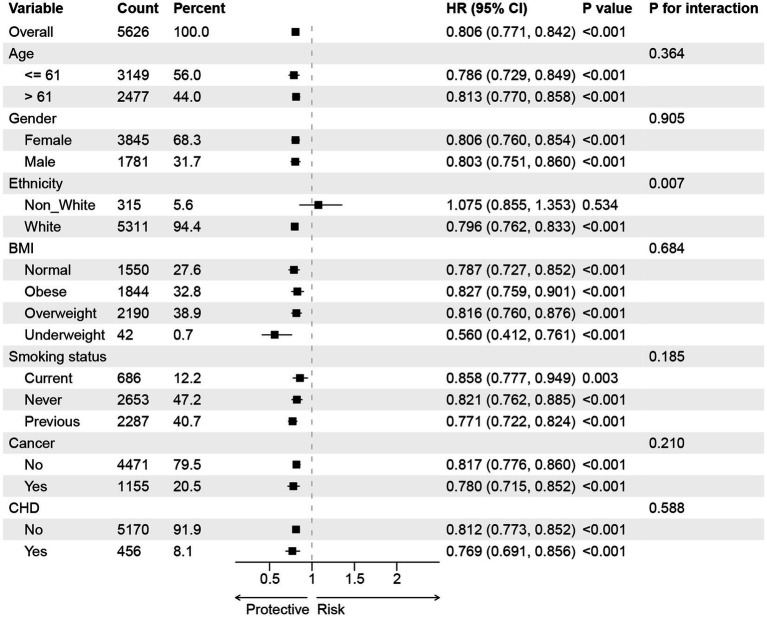
Forest plot of survival outcomes across subgroups. The forest plot illustrates the fully adjusted HRs and 95% CIs of the lnCALLY index across various prespecified baseline subgroups. Interaction *p* values were calculated using likelihood ratio tests. The results demonstrate that the protective prognostic utility of a higher lnCALLY index is generally consistent across most demographic and clinical subgroups. CHD, coronary heart disease.

The subgroup analyses generally supported the stability of the association between lnCALLY and all-cause mortality. Higher lnCALLY values were consistently associated with lower mortality risk across most subgroups. No significant interactions were detected for age, gender, BMI category, smoking status, baseline cancer status, or history of CHD, with all *p* values for interaction above 0.05 (Figure 3). In these subgroups, the HRs remained around 0.80, indicating that the prognostic association was not materially modified by these baseline characteristics or comorbidities.

Ethnicity was the only subgroup variable showing a statistically significant interaction (*p* for interaction = 0.007). Among White participants, the inverse association remained strong and statistically significant (HR = 0.796, 95% CI, 0.762 to 0.833; *p* < 0.001). In the non-White subgroup, the association did not reach statistical significance (HR = 1.075, 95% CI, 0.855 to 1.353; *p* = 0.534). Given the small number of non-White participants, who accounted for only 5.6% of the cohort (*n* = 315), this result is more likely to reflect imprecision and limited power than a definitive difference in prognostic relevance.

### Incremental predictive value and clinical utility of the CALLY index

**Figure 4:**
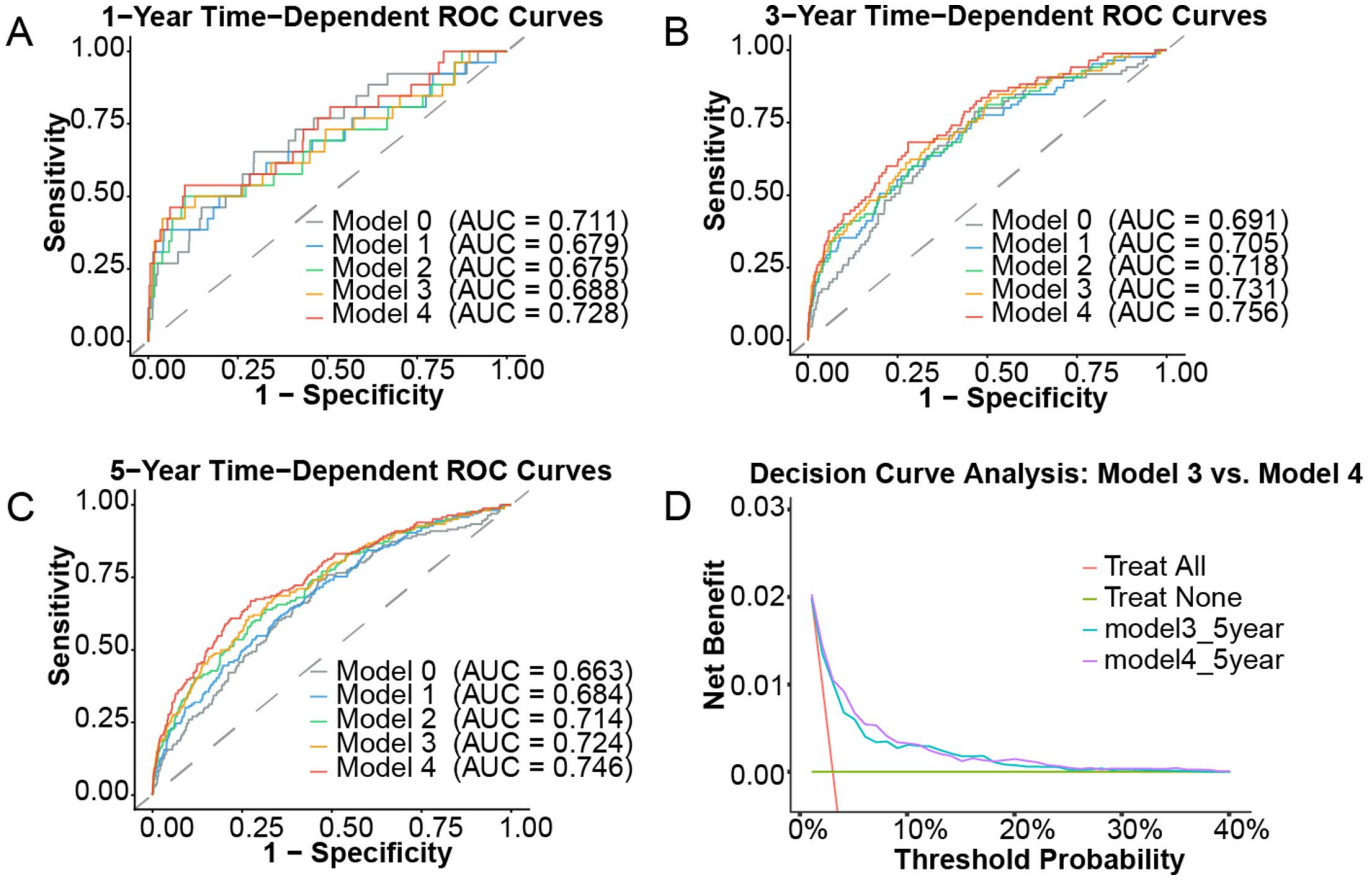
Predictive performance of the models incorporating the CALLY index versus the clinical baseline model. Time-dependent ROC curves comparing the discriminative ability of five models (Model 0–4) at 1-year **(A)**, 3-year **(B)**, and 5-year **(C)** follow-up. **(D)** Decision curve analysis comparing the net benefit of Model 3 and Model 4 at 5 years. Model 4 (purple line) provides a higher net benefit than Model 3 (cyan line), indicating its improved clinical utility.

To evaluate the incremental predictive value of the CALLY index, model performance was compared at 1, 3, and 5 years. Adding comprehensive laboratory parameters to the baseline clinical model improved prognostic accuracy, as shown by the comparison between Model 3 and Model 4 (Figures 4A–C). At 5 years, the AUC increased from 0.724 in Model 3 to 0.746 in Model 4. Reclassification performance also improved, with significant gains in IDI (*p* < 0.001) and NRI (*p* < 0.001; Supplementary Table S8). DCA further supported the potential clinical utility of Model 4 (Figure 4D). Although the absolute net benefit difference between Model 3 and Model 4 was modest, Model 4 provided additional net benefit in a broad range of clinically relevant threshold probabilities (Supplementary Table S9). Because the added biomarkers, including AST, ALT, LDL, and creatinine, are inexpensive and routinely available in clinical practice, these modest improvements may still be meaningful for refining risk stratification in patients with RA.

### Sensitivity analysis results

The association was re-examined in the original non-imputed dataset. The inverse relationship between the lnCALLY index and all-cause mortality remained statistically significant, with a fully adjusted HR of 0.808 (95% CI, 0.772 to 0.846, *p* < 0.001; Supplementary Table S10), closely matching that of the imputed dataset (HR = 0.806). To assess the potential influence of extreme lymphocyte values, the Cox regression analyses were then repeated after excluding participants with lymphocyte count > 5 × 10^9/L and, separately, after excluding values above Q3 + 3IQR. The association remained essentially unchanged in the fully adjusted model after excluding lymphocyte count > 5 × 10^9/L (HR = 0.803, 95% CI, 0.769 to 0.840, *p* < 0.001) and after excluding values above Q3 + 3IQR (HR = 0.803, 95% CI, 0.768 to 0.839, *p* < 0.001; Supplementary Table S11). To reduce the possibility of reverse causality, participants who died within the first year of follow-up were further excluded. The protective association of lnCALLY persisted after this exclusion, although the effect estimate was slightly attenuated (HR = 0.812, 95% CI, 0.777 to 0.849, *p* < 0.001; Supplementary Table S12). The dose–response pattern was also tested using an alternative categorization based on lnCALLY tertiles. Compared with participants in the lowest tertile, those in the middle and highest tertiles had progressively lower risks of all-cause mortality, with HRs of 0.640 and 0.521, respectively (*p* for trend < 0.001; Supplementary Table S13). Taken together, these sensitivity analyses consistently supported an independent inverse association between the lnCALLY index and all-cause mortality in patients with RA.

## Discussion

In this large prospective cohort of patients with RA, higher CALLY index values were independently associated with a lower risk of all-cause mortality. This relationship showed a graded inverse pattern, and this pattern was broadly consistent across demographic and clinical subgroups. These findings support the CALLY index as a simple composite marker that may help refine mortality risk assessment in patients with RA.

The biological basis for this association is plausible, because the CALLY index integrates inflammation, nutrition, and immune status. This composite nature may explain why CALLY has shown prognostic value across several diseases (12–15). In RA specifically, its correlation with disease activity further supports its relevance for clinical risk assessment (16). Each component of the index reflects a key aspect of RA pathophysiology. CRP reflects persistent systemic inflammation, a process driven in part by proinflammatory cytokines that contribute not only to synovial injury but also to extra-articular vascular complications (17). Albumin may indicate nutritional reserve and systemic illness burden. Low albumin levels are often linked to rheumatoid cachexia, sarcopenic obesity, disability, and adverse cardiovascular outcomes (18, 19). Lymphocyte count provides information on immune homeostasis. In active RA, lymphopenia may arise from sustained immune activation and cytokine-mediated apoptosis (20, 21). These features suggest that a higher CALLY value may represent a more favorable biological state, namely lower inflammatory activity, better nutritional reserve, and relatively preserved immune function (22, 23).

Our findings in this European cohort align with previous evidence from American populations (10). Similar HRs and inflection points across cohorts suggest that the prognostic value of CALLY may not be restricted to a single clinical setting. Still, the subgroup results need careful interpretation. The inverse association was clear among White participants but did not reach statistical significance in the non-White subgroup, likely reflecting limited sample size and lower statistical power. This result should not be read as evidence that CALLY lacks value in non-White patients. Instead, it points to the need for validation in larger and more ethnically diverse RA cohorts.

The graded inverse association between CALLY and mortality may have clinical value. In this study, mortality risk declined progressively across increasing CALLY quartiles, suggesting that the index could help support routine risk stratification in patients with RA. Since CALLY is calculated from inexpensive and widely available laboratory measures, it is feasible to incorporate into outpatient follow-up without increasing cost. Patients with persistently low CALLY values may deserve closer evaluation, particularly for inflammatory control, nutritional status, and cardiovascular risk. However, this point should be interpreted cautiously. The present observational design cannot determine whether actively improving CALLY values would translate into better survival outcomes.

Several limitations should be considered. First, our analysis relied only on baseline CALLY values, which prevented us from assessing whether changes in the index over time carried additional prognostic information. Moreover, the prognostic value of baseline CALLY weakened over the full follow-up, eventually violating the proportional hazards assumption. Since dynamic immune-nutritional markers naturally fluctuate, this decay is biologically plausible. It suggests that baseline CALLY is most clinically relevant within a relatively short term. Second, RA cases were identified using registry algorithms and electronic health records, minor disease misclassification therefore cannot be excluded. Third, because of the observational design, causal inference cannot be established. Residual confounding is also possible, as several RA-specific variables were not fully available in this large population-based cohort. Specifically, disease duration and detailed pharmacotherapy history, including cumulative corticosteroid exposure and biologic DMARD use, were not counted. The observed association may therefore partly reflect unmeasured variation in disease severity or treatment intensity. However, a recent clinical cohort of 1,058 patients with RA reported that the CALLY index remained independently associated with prognosis after adjustment for disease duration, glucocorticoid use, and biologic therapies (24). This finding supports the potential relevance of the index, although prospective validation in dedicated RA cohorts remains necessary.

Before the CALLY index can be more widely applied, its standardized cut-off values and clinical use criteria need to be prospectively validated. Future studies should test its performance in clinically diverse populations, including patients with metabolic disorders, older adults, and individuals receiving different treatment regimens. Its discriminatory ability may also be improved by integrating CALLY with other emerging biomarkers, such as gut microbiota. Besides, longitudinal studies are needed to determine whether changes in CALLY over time provide additional prognostic information (25, 26). Such studies should also examine cause-specific mortality and explore whether interventions targeting related inflammatory or metabolic pathways can translate into better clinical outcomes.

## Data Availability

The datasets presented in this study can be found in online repositories. The names of the repository/repositories and accession number(s) can be found in the article/Supplementary material.
