# Supplementary material for: Association between CALLY index and all-cause mortality in patients with rheumatoid arthritis
Source: Front Nutr. 2026 Jun 16;13:1829076. doi: 10.3389/fnut.2026.1829076 (PMC13317069; doi:10.3389/fnut.2026.1829076)
Supplement: Supplementary file 1 [file Data_Sheet_1.PDF]

Supplementary materials

Table S1 Hazard ratios and 95% confidence intervals for the core subset of clinical predictors retained by the AIC algorithm.

**Variable**	**Hazard Ratio**	**Lower 95% CI**	**Upper 95% CI**	***P* Value**
lnCALLY	0.685	0.612	0.767	< 0.001
Age	1.048	1.019	1.077	< 0.001
Ethnicity=White	2.457	0.881	6.856	0.086
BMI=Obese	0.695	0.437	1.106	0.125
BMI=Overweight	1.384	0.931	2.058	0.108
BMI=Underweight	2	0.607	6.592	0.255
Education=Other	0.946	0.593	1.51	0.816
Education=UnKnown	1.349	0.844	2.155	0.211
Smoking status=Never	0.326	0.212	0.502	< 0.001
Smoking status=Previous	0.516	0.348	0.767	0.001
Cancerstatus=Yes	1.381	0.983	1.939	0.063
CHD=Yes	2.045	1.378	3.036	< 0.001
ALT	0.982	0.964	1	0.056
AST	1.021	1.004	1.038	0.015
LDL	0.859	0.714	1.032	0.105
HbA1c	1.039	1.025	1.054	< 0.001
Creatinine	1.006	1.002	1.011	0.007

Abbreviations: AIC, Akaike Information Criterion; CI, confidence interval; BMI, body mass index; CHD, coronary heart disease; ALT, alanine aminotransferase.

Note: This table presents the optimal parsimonious model retaining the core subset of predictors from an initial pool of 25 candidate clinical variables, selected via a rigorous stepwise algorithm minimizing the AIC. For categorical variables, the unlisted category serves as the reference group (e.g., "Normal" for BMI, "No" for Cancer status and CHD).

Table S2 Characteristics of Study Participants According to lnCALLY Quartiles.

	Excluded	Included	*P*	SMD
n	1193	5626		
Age (median [IQR])	61.00 [54.00, 65.00]	61.00 [55.00, 65.00]	0.642	0.022
Gender = Male (%)	349 (29.3)	1781 (31.7)	0.111	0.052
Ethnicity = White (%)	1114 (93.7)	5303 (94.4)	0.368	0.03
BMI (%)			0.663	0.039
Normal (>=18.5 <25)	320 (27.4)	1535 (27.5)		
Obese (>=30)	392 (33.6)	1827 (32.7)		
Overweight (>=25 <30)	442 (37.9)	2176 (39.0)		
Underweight (<18.5 ~ 1)	12 (1.0)	41 (0.7)		
Education (%)			0.67	0.028
College	254 (21.3)	1233 (21.9)		
Other	572 (47.9)	2734 (48.6)		
UnKnown	367 (30.8)	1659 (29.5)		
Smoking status (%)			0.083	0.071
Current	164 (13.9)	679 (12.2)		
Never	569 (48.3)	2626 (47.1)		
Previous	444 (37.7)	2274 (40.8)		
Cancer = Yes (%)	245 (20.5)	1155 (20.5)	1	<0.001
CHD[a] = Yes (%)	116 (9.7)	456 (8.1)	0.076	0.057
ALT (median [IQR])	19.94 [15.12, 27.04]	20.04 [15.23, 27.16]	0.904	0.007
AST (median [IQR])	24.75 [21.37, 29.50]	24.70 [21.00, 29.40]	0.527	0.032

LDL (median [IQR])	3.42 [2.80, 4.08]	3.44 [2.86, 4.06]	0.48	0.026
HbA1c (median [IQR])	35.30 [32.40, 38.50]	35.40 [32.50, 38.40]	0.79	0.001
Creatinine (median [IQR])	66.80 [56.95, 79.00]	67.00 [58.50, 77.80]	0.379	0.016

[a]CHD, chronic heart disease

Table S3 Baseline Characteristics by Survival Outcome.

Characteristic	Live	Dead	*P*
Age (median [IQR])	60.00 (53.00, 64.00)	64.00 (60.00, 67.00)	<0.001
Gender = Male (%)	1,288.00 (28.96%)	493.00 (41.82%)	<0.001
Ethnicity = White (%)	4,187.00 (94.15%)	1,124.00 (95.34%)	0.134
BMI			0.001
Normal (>=18.5 <25)	1,247.00 (28.04%)	303.00 (25.70%)	
Obese (>=30)	1,434.00 (32.25%)	410.00 (34.78%)	
Overweight (>=25 <30)	1,742.00 (39.17%)	448.00 (38.00%)	
Underweight (<18.5 ~ 1)	24.00 (0.54%)	18.00 (1.53%)	
Education			<0.001
College	1,034.00 (23.25%)	199.00 (16.88%)	
Other	2,232.00 (50.19%)	502.00 (42.58%)	
UnKnown	1,181.00 (26.56%)	478.00 (40.54%)	
Smoking status			<0.001
Current	458.00 (10.30%)	228.00 (19.34%)	
Never	2,247.00 (50.53%)	406.00 (34.44%)	
Previous	1,742.00 (39.17%)	545.00 (46.23%)	

Characteristic	Live	Dead	*P*
Cancer = Yes (%)	850.00 (19.11%)	305.00 (25.87%)	<0.001
CHD[a] = Yes (%)	271.00 (6.09%)	185.00 (15.69%)	<0.001
ALT (median [IQR])	20.20 (15.35, 27.38)	19.35 (14.86, 26.26)	0.006
AST (median [IQR])	24.60 (21.00, 29.20)	25.00 (21.10, 30.40)	0.062
LDL (median [IQR])	3.49 (2.92, 4.10)	3.24 (2.61, 3.95)	<0.001
HbA1c (median [IQR])	35.10 (32.30, 38.10)	36.20 (33.00, 40.30)	<0.001
Creatinine (median [IQR])	66.30 (58.10, 76.60)	70.50 (60.10, 82.70)	<0.001
CRP (median [IQR])	2.45 (1.17, 5.63)	3.85 (1.71, 8.97)	<0.001
Albumin (median [IQR])	44.60 (42.84, 46.41)	43.68 (41.59, 45.58)	<0.001
Lymphocyte (median [IQR])	1.80 (1.40, 2.22)	1.70 (1.30, 2.21)	<0.001
lnCALLY (median [IQR])	1.15 (0.32, 1.98)	0.68 (-0.25, 1.57)	<0.001

[a]CHD, chronic heart disease

Table S4 Variance inflation factor (VIF) analysis for covariates included in the fully adjusted Cox proportional hazards model.

Variable	GVIF
lnCALLY	1.031
Age	1.111
Gender	1.237
Ethnicity	1.071
BMI	1.259
Education	1.090
Smoking status	1.118
Cancer	1.025
CHD	1.151
ALT	3.199
AST	2.963
LDL	1.171
HbA1c	1.151
Creatinine	1.156

Table S5 Verification of the proportional hazards assumption using the Schoenfeld residuals test for the fully adjusted Cox model.

Variable	χ^2 (5-year)	*P* (5-year)	χ^2 (full follow-up)	*P* (full follow-up)
lnCALLY	2.446	0.118	5.739	0.017
Age	0.093	0.76	8.783	0.003
Gender	1.017	0.313	0.483	0.487
Ethnicity	0.148	0.701	0.010	0.919
BMI	1.584	0.663	5.306	0.151
Education	0.088	0.957	0.230	0.892
Smoking	3.101	0.212	2.261	0.323
Cancer	0.518	0.472	0.583	0.445
CHD	0.617	0.432	0.683	0.409
ALT	0.71	0.399	1.026	0.311
AST	2.365	0.124	1.199	0.274
LDL	0.204	0.651	3.683	0.055
HbA1c	0.013	0.91	0.219	0.640
Creatinine	0.722	0.395	0.362	0.548
Global	13.563	0.757	34.231	0.012

Table S6 IIncremental prognostic value of the lnCALLY index compared to its separate components and the multi-component model.

Comparison	Time	ΔAUC (95%CI)	*P* value	IDI (95% CI)	*P* value (IDI)	NRI (95% CI)	*P* value (NRI)
**lnCALLY vs. CRP**	1-year	-0.036	0.339	-0.001 (-0.004 - 0.000)	0.206	0.086 (-0.100 - 0.280)	0.312
	3-year	0.006	0.736	0.000 (-0.003 - 0.002)	0.651	0.233 (0.124 - 0.319)	<0.001
	5-year	0.005	0.678	0.001 (-0.002 - 0.004)	0.359	0.173 (0.094 - 0.250)	<0.001
**lnCALLY vs. Albumin**	1-year	-0.025	0.695	-0.001 (-0.002 - 0.000)	0.239	-0.081 (-0.265 - 0.133)	0.439
	3-year	0.029	0.412	-0.000 (-0.002 - 0.002)	0.718	0.038 (-0.090 - 0.157)	0.505
	5-year	0.044	0.075	0.000 (-0.003 - 0.003)	0.957	0.046 (-0.065 - 0.139)	0.352
**lnCALLY vs. Lymphocyte**	1-year	0.052	0.407	0.001 (-0.029 - 0.002)	0.306	0.270 (0.102 - 0.415)	0.007
	3-year	0.085	0.020	0.003 (-0.021 - 0.004)	0.153	0.305 (0.204 - 0.385)	<0.001
	5-year	0.100	<0.001	0.005 (-0.011 - 0.007)	0.120	0.267 (0.177 - 0.330)	<0.001
**CALLY model vs. multi-component model**	1-year	-0.011	0.508	-0.001 (-0.110 - 0.001)	0.385	-0.046 (-0.225 - 0.223)	0.997

	3-year	0.000	0.957	0.000 (-0.040 - 0.004)	0.999	-0.037 (-0.146 - 0.144)	0.999
	5-year	0.005	0.428	0.001 (-0.019 - 0.004)	0.930	0.048 (-0.044 - 0.141)	0.352

Table S7 Pairwise post hoc log-rank tests with Bonferroni correction for the overall survival data.

	**Q1 [-3.87, 0.187]**	**Q2 (0.187, 1.05]**	**Q3 (1.05, 1.92]**
**Q2 (0.187, 1.05]**	<0.001	NA	NA
**Q3 (1.05, 1.92]**	<0.001	0.058	NA
**Q4 (1.92, 4.79]**	<0.001	<0.001	0.952

Table S8 Incremental discrimination and risk reclassification performance of Model 4 compared with Model 3 for predicting all-cause mortality.

Time	Model 3 AUC	Model 4 AUC	ΔAUC	*P* value (ΔAUC)	IDI (95% CI)	*P* value (IDI)	NRI (95% CI)	*P* value (NRI)
1-year	0.688	0.728	0.04	0.195	0.005 (0.001-0.013)	0.007	0.214 (0.058-0.424)	0.02
3-year	0.731	0.756	0.025	0.042	0.006 (0.001-0.014)	0.007	0.273 (0.144-0.380)	<0.001
5-year	0.724	0.746	0.022	0.004	0.008 (0.003-0.016)	<0.001	0.189 (0.086-0.284)	<0.001

Table S9 Decision curve analysis metrics and added clinical benefit per 1,000 patients for 5-year mortality prediction.

Threshold Probability	Treat All	Treat None	Model 3 (Net Benefit)	Model 4 (Net Benefit)	ΔNet Benefit (M4-M3)	Added Benefit per 1000 Pts
0.05	-0.021	0.000	0.006	0.007	0.001	0.739
0.1	-0.078	0.000	0.003	0.003	0.000	0.158
0.15	-0.142	0.000	0.002	0.001	-0.001	-0.565
0.2	-0.213	0.000	0.001	0.002	0.001	0.711
0.3	-0.386	0.000	0.000	0.000	0.000	0.102
0.4	-0.617	0.000	0.000	0.000	0.000	0.059

Table S10 Cox Regression Model Results for lnCALLY and All-Cause Mortality (No Multiple Imputation).

Variable	Model 0 HR (95% CI)	Model 1 HR (95% CI)	Model 2 HR (95% CI)	Model 3 HR (95% CI)	Model 4 HR (95% CI)
**Lymphocyte (continuous)**	0.983 (0.904-1.069)	1.030 (0.957-1.107)	0.958 (0.881-1.043)	0.953 (0.875-1.039)	0.964 (0.885-1.050)
**Lymphocyte (4-class)**					
[0.22,1.4]	Ref	Ref	Ref	Ref	Ref
(1.4,1.79]	0.638 (0.539-0.756)	0.685 (0.578-0.811)	0.668 (0.564-0.791)	0.664 (0.561-0.787)	0.675 (0.569-0.800)
(1.79,2.22]	0.682 (0.580-0.801)	0.750 (0.638-0.882)	0.699 (0.594-0.823)	0.691 (0.587-0.814)	0.694 (0.589-0.819)
(2.22,30.2]	0.767 (0.656-0.897)	0.866 (0.740-1.014)	0.733 (0.622-0.862)	0.718 (0.609-0.845)	0.723 (0.613-0.853)
P for trend	<0.001	0.067	<0.001	<0.001	<0.001
**CRP (continuous)**	1.022 (1.017-1.027)	1.020 (1.015-1.025)	1.018 (1.013-1.023)	1.018 (1.013-1.023)	1.017 (1.012-1.022)
**CRP (4-class)**					
[0.09,1.25]	Ref	Ref	Ref	Ref	Ref
(1.25,2.69]	1.128 (0.934-1.362)	1.035 (0.857-1.249)	1.008 (0.833-1.220)	1.022 (0.844-1.237)	1.042 (0.861-1.261)
(2.69,6.16]	1.441 (1.204-1.724)	1.368 (1.143-1.637)	1.314 (1.094-1.579)	1.332 (1.109-1.600)	1.346 (1.120-1.617)
(6.16,77.67]	2.074 (1.751-2.457)	1.968 (1.661-2.332)	1.808 (1.520-2.151)	1.837 (1.544-2.186)	1.820 (1.529-2.165)
P for trend	<0.001	<0.001	<0.001	<0.001	<0.001

**Albumin (continuous)**	0.892 (0.875-0.911)	0.897 (0.879-0.916)	0.904 (0.886-0.923)	0.905 (0.886-0.923)	0.911 (0.892-0.930)
**Albumin (4-class)**					
[27.12,42.64]	Ref	Ref	Ref	Ref	Ref
(42.64,44.44]	0.652 (0.559-0.761)	0.653 (0.559-0.762)	0.655 (0.561-0.765)	0.661 (0.566-0.772)	0.698 (0.597-0.816)
(44.44,46.27]	0.571 (0.486-0.670)	0.586 (0.499-0.688)	0.605 (0.515-0.711)	0.603 (0.513-0.708)	0.632 (0.537-0.744)
(46.27,55.44]	0.452 (0.380-0.536)	0.477 (0.401-0.568)	0.496 (0.417-0.591)	0.493 (0.415-0.587)	0.516 (0.432-0.615)
P for trend	<0.001	<0.001	<0.001	<0.001	<0.001
**lnCALLY (continuous)**	0.775 (0.741-0.811)	0.795 (0.760-0.832)	0.801 (0.766-0.839)	0.801 (0.765-0.838)	0.808 (0.772-0.846)
**lnCALLY (4-class)**					
[-3.872,0.1919]	Ref	Ref	Ref	Ref	Ref
(0.1919,1.059]	0.622 (0.533-0.725)	0.631 (0.541-0.736)	0.637 (0.546-0.743)	0.629 (0.539-0.734)	0.652 (0.558-0.761)
(1.059,1.922]	0.500 (0.425-0.589)	0.516 (0.438-0.607)	0.527 (0.447-0.621)	0.516 (0.438-0.609)	0.528 (0.448-0.623)
(1.922,4.786]	0.444 (0.375-0.525)	0.480 (0.405-0.569)	0.499 (0.420-0.592)	0.490 (0.413-0.582)	0.505 (0.425-0.600)
P for trend	<0.001	<0.001	<0.001	<0.001	<0.001

Table S11 Sensitivity analysis excluding participants with extreme lymphocyte values**.**

Scenario	Exclusion rule	N Excluded	Model	N used	HR (95%CI)	*P*
Full sample	No exclusion	0	Model 0	5626	0.775 (0.742-0.809)	<0.001
Full sample	No exclusion	0	Model 1	5626	0.795 (0.761-0.830)	<0.001
Full sample	No exclusion	0	Model 2	5626	0.800 (0.765-0.836)	<0.001
Full sample	No exclusion	0	Model 3	5626	0.799 (0.765-0.835)	<0.001
Full sample	No exclusion	0	Model 4	5626	0.806 (0.771-0.842)	<0.001
Exclude lymphocyte > Q3+3IQR	Lymphocyte <= 4.68×10^9/L	21	Model 0	5605	0.772 (0.738-0.806)	<0.001
Exclude lymphocyte > Q3+3IQR	Lymphocyte <= 4.68×10^9/L	21	Model 1	5605	0.792 (0.758-0.827)	<0.001
Exclude lymphocyte > Q3+3IQR	Lymphocyte <= 4.68×10^9/L	21	Model 2	5605	0.797 (0.762-0.833)	<0.001
Exclude lymphocyte > Q3+3IQR	Lymphocyte <= 4.68×10^9/L	21	Model 3	5605	0.796 (0.762-0.832)	<0.001
Exclude lymphocyte > Q3+3IQR	Lymphocyte <= 4.68×10^9/L	21	Model 4	5605	0.803 (0.768-0.839)	<0.001
Exclude lymphocyte > 5	Lymphocyte <= 5.00×10^9/L	14	Model 0	5612	0.773 (0.740-0.807)	<0.001
Exclude lymphocyte > 5	Lymphocyte <= 5.00×10^9/L	14	Model 1	5612	0.793 (0.759-0.828)	<0.001
Exclude lymphocyte > 5	Lymphocyte <= 5.00×10^9/L	14	Model 2	5612	0.797 (0.763-0.833)	<0.001
Exclude lymphocyte > 5	Lymphocyte <= 5.00×10^9/L	14	Model 3	5612	0.797 (0.763-0.833)	<0.001
Exclude lymphocyte > 5	Lymphocyte <= 5.00×10^9/L	14	Model 4	5612	0.803 (0.769-0.840)	<0.001
Exclude lymphocyte > 10	Lymphocyte <= 10.00×10^9/L	4	Model 0	5622	0.772 (0.739-0.806)	<0.001

Exclude lymphocyte > 10	Lymphocyte <= 10.00×10^9/L	4	Model 1	5622	0.792 (0.758-0.828)	<0.001
Exclude lymphocyte > 10	Lymphocyte <= 10.00×10^9/L	4	Model 2	5622	0.797 (0.762-0.832)	<0.001
Exclude lymphocyte > 10	Lymphocyte <= 10.00×10^9/L	4	Model 3	5622	0.796 (0.762-0.832)	<0.001
Exclude lymphocyte > 10	Lymphocyte <= 10.00×10^9/L	4	Model 4	5622	0.803 (0.768-0.839)	<0.001

Table S12 Cox Regression Results for the Association Between lnCALLY and All-Cause Mortality (Excluding Participants Who Died Within the First Year of Follow-Up).

**Variable**	**Model 0 HR (95% CI)**	**Model 1 HR (95% CI)**	**Model 2 HR (95% CI)**	**Model 3 HR (95% CI)**	**Model 4 HR (95% CI)**
**Lymphocyte (continuous)**	0.962 (0.884-1.046)	1.009 (0.935-1.088)	0.927 (0.851-1.011)	0.921 (0.844-1.004)	0.926 (0.849-1.010)
**Lymphocyte (4-class)**					
[0.22,1.4]	Ref	Ref	Ref	Ref	Ref
(1.4,1.79]	0.672 (0.570-0.792)	0.712 (0.604-0.839)	0.689 (0.585-0.812)	0.688 (0.583-0.811)	0.689 (0.584-0.813)
(1.79,2.22]	0.704 (0.601-0.825)	0.770 (0.657-0.902)	0.714 (0.609-0.838)	0.706 (0.602-0.829)	0.705 (0.601-0.828)
(2.22,18.5]	0.795 (0.682-0.926)	0.892 (0.764-1.040)	0.748 (0.638-0.878)	0.733 (0.625-0.860)	0.731 (0.622-0.858)
P for trend	0.003	0.13	<0.001	<0.001	<0.001
**CRP (continuous)**	1.021 (1.016-1.026)	1.020 (1.015-1.025)	1.018 (1.013-1.023)	1.018 (1.013-1.023)	1.017 (1.012-1.022)
**CRP (4-class)**					
[0.09,1.24]	Ref	Ref	Ref	Ref	Ref
(1.24,2.69]	1.152 (0.958-1.385)	1.039 (0.864-1.250)	1.018 (0.845-1.227)	1.034 (0.858-1.246)	1.055 (0.875-1.271)
(2.69,6.21]	1.468 (1.231-1.750)	1.382 (1.158-1.648)	1.333 (1.113-1.596)	1.349 (1.127-1.616)	1.376 (1.149-1.647)
(6.21,77.67]	2.068 (1.750-2.442)	1.934 (1.637-2.285)	1.796 (1.514-2.130)	1.824 (1.537-2.164)	1.815 (1.529-2.153)
P for trend	<0.001	<0.001	<0.001	<0.001	<0.001

**Albumin (continuous)**	0.893 (0.876-0.911)	0.899 (0.881-0.917)	0.905 (0.887-0.923)	0.905 (0.887-0.924)	0.912 (0.894-0.931)
**Albumin (4-class)**					
[27.12,42.59]	Ref	Ref	Ref	Ref	Ref
(42.59,44.41]	0.645 (0.555-0.751)	0.646 (0.555-0.752)	0.644 (0.553-0.749)	0.652 (0.560-0.759)	0.693 (0.594-0.807)
(44.41,46.27]	0.569 (0.486-0.665)	0.582 (0.497-0.681)	0.597 (0.510-0.699)	0.597 (0.510-0.699)	0.630 (0.537-0.739)
(46.27,55.44]	0.456 (0.386-0.540)	0.489 (0.413-0.579)	0.507 (0.428-0.601)	0.504 (0.425-0.598)	0.529 (0.445-0.628)
P for trend	<0.001	<0.001	<0.001	<0.001	<0.001
**lnCALLY (continuous)**	0.782 (0.748-0.817)	0.802 (0.768-0.839)	0.807 (0.772-0.844)	0.807 (0.772-0.843)	0.812 (0.777-0.849)
**lnCALLY (4-class)**					
[-3.456,0.1919]	Ref	Ref	Ref	Ref	Ref
(0.1919,1.057]	0.636 (0.547-0.740)	0.648 (0.557-0.753)	0.654 (0.562-0.760)	0.643 (0.553-0.748)	0.666 (0.573-0.775)
(1.057,1.918]	0.519 (0.442-0.608)	0.534 (0.455-0.626)	0.543 (0.463-0.638)	0.533 (0.454-0.626)	0.542 (0.461-0.636)
(1.918,4.786]	0.454 (0.384-0.535)	0.494 (0.419-0.583)	0.508 (0.430-0.601)	0.500 (0.423-0.592)	0.512 (0.433-0.607)
P for trend	<0.001	<0.001	<0.001	<0.001	<0.001

Table S13 Cox Regression Results for the Association Between lnCALLY Tertiles and All-Cause Mortality (vs. Lowest Tertile as Reference).

Variable	Model 0 HR (95% CI)	Model 1 HR (95% CI)	Model 2 HR (95% CI)	Model 3 HR (95% CI)	Model 4 HR (95% CI)
**Lymphocyte (continuous)**	0.983 (0.904-1.069)	1.030 (0.957-1.107)	0.958 (0.881-1.043)	0.953 (0.875-1.039)	0.964 (0.885-1.050)
**Lymphocyte (3-class)**					
[0.22,1.5] (T1)	Ref	Ref	Ref	Ref	Ref
(1.5,2.06] (T2)	0.727 (0.629-0.840)	0.797 (0.689-0.921)	0.777 (0.672-0.900)	0.776 (0.671-0.898)	0.776 (0.670-0.898)
(2.06,30.2] (T3)	0.800 (0.694-0.921)	0.902 (0.782-1.039)	0.782 (0.675-0.907)	0.768 (0.663-0.891)	0.768 (0.662-0.892)
P for trend	0.001	0.118	<0.001	<0.001	<0.001
**CRP (continuous)**	1.022 (1.017-1.027)	1.020 (1.015-1.025)	1.018 (1.013-1.023)	1.018 (1.013-1.023)	1.017 (1.012-1.022)
**CRP (3-class)**					
[0.09,1.66] (T1)	Ref	Ref	Ref	Ref	Ref
(1.66,4.6] (T2)	1.263 (1.076-1.483)	1.203 (1.025-1.413)	1.189 (1.010-1.399)	1.205 (1.024-1.418)	1.217 (1.034-1.432)
(4.6,77.67] (T3)	2.021 (1.743-2.343)	1.982 (1.709-2.298)	1.849 (1.589-2.152)	1.866 (1.603-2.172)	1.847 (1.587-2.151)
P for trend	<0.001	<0.001	<0.001	<0.001	<0.001
**Albumin (continuous)**	0.892 (0.875-0.911)	0.897 (0.879-0.916)	0.904 (0.886-0.923)	0.905 (0.886-0.923)	0.911 (0.892-0.930)
**Albumin (3-class)**					

[27.12,43.27] (T1)	Ref	Ref	Ref	Ref	Ref
(43.27,45.62] (T2)	0.661 (0.575-0.759)	0.669 (0.582-0.769)	0.680 (0.592-0.781)	0.669 (0.582-0.768)	0.697 (0.606-0.802)
(45.62,55.44] (T3)	0.521 (0.449-0.604)	0.553 (0.477-0.643)	0.576 (0.496-0.669)	0.573 (0.493-0.665)	0.595 (0.511-0.693)
P for trend	<0.001	<0.001	<0.001	<0.001	<0.001
**lnCALLY (continuous)**	0.775 (0.741-0.811)	0.795 (0.760-0.832)	0.801 (0.766-0.839)	0.801 (0.765-0.838)	0.808 (0.772-0.846)
**lnCALLY (3-class)**					
[-3.872,0.5178] (T1)	Ref	Ref	Ref	Ref	Ref
(0.5178,1.615] (T2)	0.616 (0.537-0.708)	0.626 (0.545-0.719)	0.637 (0.555-0.732)	0.622 (0.541-0.715)	0.640 (0.557-0.736)
(1.615,4.786] (T3)	0.479 (0.413-0.556)	0.503 (0.433-0.584)	0.525 (0.451-0.610)	0.515 (0.442-0.599)	0.521 (0.448-0.606)
P for trend	<0.001	<0.001	<0.001	<0.001	<0.001

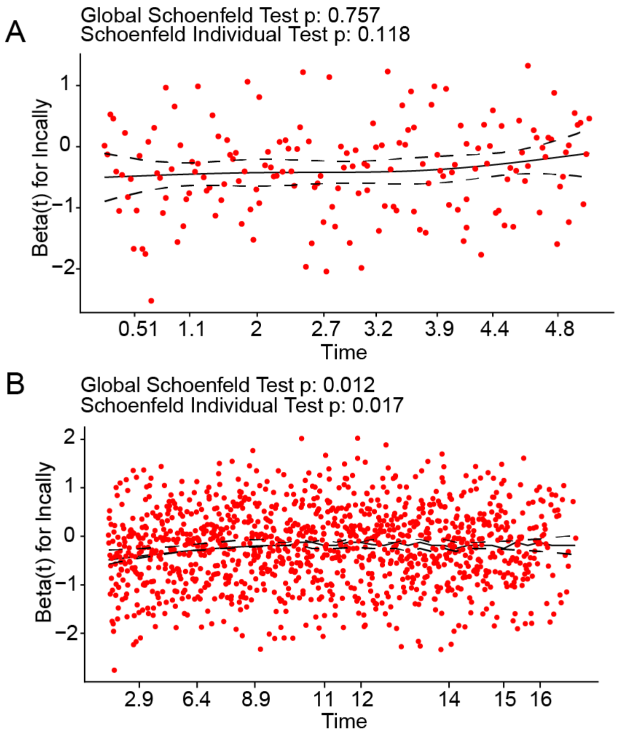

Fig.S1 Scaled Schoenfeld residual plots for the lnCALLY index.

(A) Within the primary 5-year follow-up window, the proportional hazards assumption was satisfied (Global $P = 0.757$, lnCALLY $P = 0.118$). **(B)** Over the full follow-up period, the PH assumption was not met (Global $P = 0.012$, lnCALLY $P = 0.017$), reflecting the time-dependent attenuation of the baseline biomarker's predictive capacity. In both panels, the solid line represents the smoothing spline fit, and the dashed lines indicate the 95% confidence intervals.

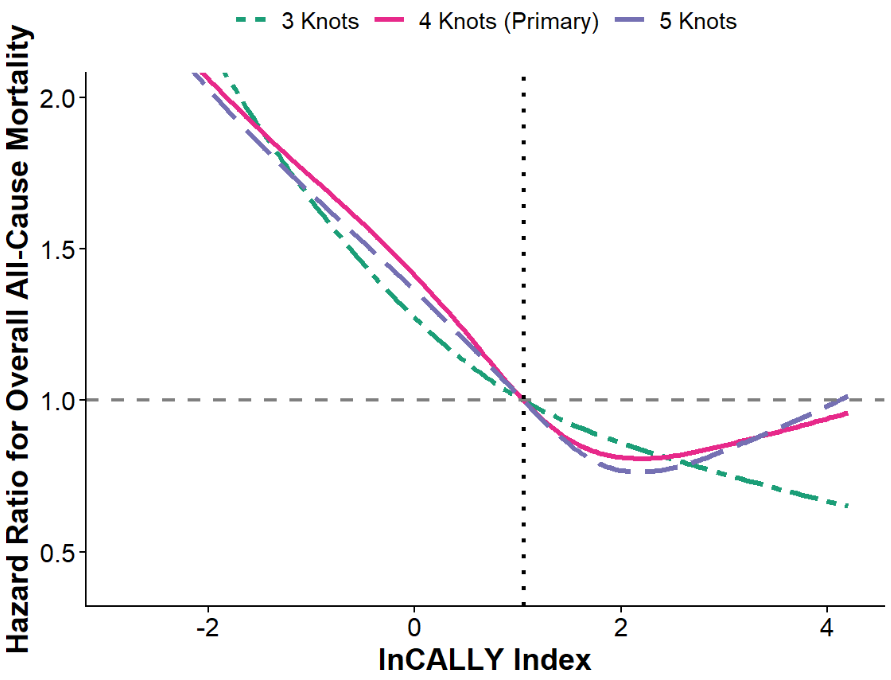

Fig. S2 Sensitivity analysis of restricted cubic spline (RCS) knot placement.

The curves represent the estimated hazard ratios for 5-year all-cause mortality across the continuous lnCALLY index, fitted with varying knot specifications in the fully adjusted model (Model 4). The solid pink line represents the primary analysis utilizing 4 knots. The dashed green and purple lines represent alternative models using 3 and 5 knots, respectively. The horizontal dashed line indicates the reference hazard ratio of 1.0. The vertical dotted line denotes the exploratory cut-off at lnCALLY = 1.054. The consistent intersection of all three curves at the reference line demonstrates that the location of this tipping point is highly robust and independent of specific knot placement.
